# Cardiac device-related endocarditis in the patient with end-stage renal failure in the course of Fabry disease

**DOI:** 10.1007/s11255-013-0493-0

**Published:** 2013-06-29

**Authors:** Anna Tomaszuk-Kazberuk, Paulina Lopatowska, Elzbieta Mlodawska, Bozena Sobkowicz, Marek Kalinowski, Jerzy Glowinski, Wlodzimierz Musial, Jolanta Malyszko

**Affiliations:** 1 Department of Cardiology, Medical University, Białystok, Poland; 2 Fresenius Medical Care Dialysis Unit, Białystok, Poland; 3 Department of Vascular Surgery and Transplantation, Medical University, Białystok, Poland; 4 Department of Nephrology, Medical University in Bialystok, Zurawia 14, 150540 Białystok, Poland

**Keywords:** Cardiac device-related endocarditis, Fabry disease, End-stage renal failure

Editor,

Fabry disease is an X-linked lysosomal storage disorder with deficiency of alpha-galactosidase activity leading to impaired organ function due to glycosphingolipids accumulation [1]. We present the first case of cardiac device-related endocarditis (CDRE) in a 53-year-old man with Fabry disease, severe hypertrophy and CKD. He had implanted dual-chamber pacemaker 5 years ago due to bradycardia-tachycardia syndrome. He was admitted to the Cardiac Intensive Care Unit due to inflammation at the device placement site. He complains of daily recurring chills and fever, which has reached 39 °C, weakness, poor exercise tolerance and dyspnea at rest.

On admission, he was in a serious condition with systolic murmur at the heart apex, radiating to the left armpit, and a grossly fluctuant subcutaneous collection with redness and warmth at the side of the generator pocket. An ECG showed atrial fibrillation and effective ventricular pacing at 70 beats per minute. Laboratory tests revealed elevated inflammatory parameters—leukocytosis (14 × 10^9^/L), CRP (251 mg/L), procalcitonin (4.62 ng/mL), eGFR 15 mL/min and creatinine 3.7 mg/dL.

**Fig. 1:**
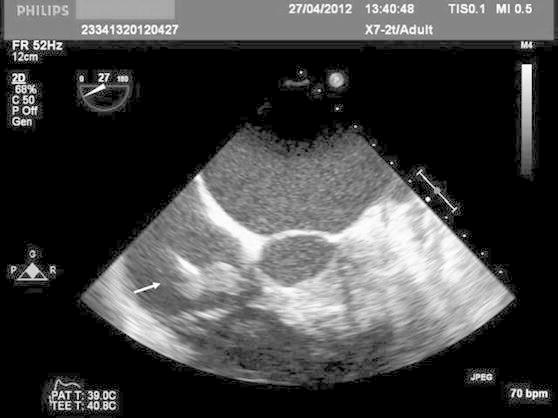
A mass in the right atrium (indicated by arrow), above the tricuspid valve, attached to the device lead on the transesophageal echocardiography (TEE)

A chest X-ray did not reveal any specific abnormalities. Ultrasonography of the area of the pacemaker pocket detected an abscess, which was then incised, drained and cultures. At the same time three blood specimens were taken for culture. A transthoracic echocardiogram (TTE) revealed left ventricular hypertrophy, ejection fraction 55 %, significant mitral regurgitation and a mobile mass of approximately 16 × 16 mm on the septal tricuspid leaflet. No presence of an abscess was recorded. A transesophageal echocardiography (TEE) revealed a mass of maximum size 12 × 22 mm in the right atrium, above the tricuspid valve, attached to the device lead (Fig. 1).

The patient was treated with parenteral vanocomycin and gentamycin according to the guidelines [2]. The microbiological results revealed Staphylococcus aureus. Due to persistent fever and signs of inflammation, rifampicin was added to the treatment. The control TEE showed no reduction in size of the bacterial vegetation.

On the 11th day of hospitalization, a percutaneous extraction of the infected pacemaker together with infected lead was performer and antibiotics were continued. After 4 weeks of therapy and obtaining negative results of blood culture, the patient had a VVI pacemaker implanted without complications. Hemodialysis was started with catheter, then the an a-v anastomosis was performed using Konner’s modification Gracz fistula [3]. After a month, a fully matured fistula was found, but over the course of the next few weeks, it became fibrotic. Additionally, edema of the left arm occurred. A CT revealed a high-grade stenosis of a segment of the cephalic vein, with outflow through collateral circulation, and complete occlusion of the subclavian vein, probably due to presented problems with pacemaker. Antegrade angiography showed a subtotal stenosis. An attempt to pass a guidewire upwards and use the retrograde approach through a higher segment of cephalic vein failed. The a–v fistula with buttonhole technique was used. Currently, the patient is on dialysis therapy on a regular basis, awaiting kidney transplantation.

We have presented an exclusive case of favorable outcome of CDRE in the patient with end-stage renal failure in the course of Fabry disease. Treatment of CDRE in Fabry patients should follow standard recommendations [2]. Surgical extraction may be considered in patients with very large (> 25 mm) vegetations [4].

The presented case is of interest for both cardiologists and nephrologists. Fabry disease is a progressive, multi-organ disease affecting mostly heart and kidneys. Cardiac and renal manifestations are the main causes of premature death in affected patients. Furthermore, many patients develop kidney failure and require renal replacement therapy before being diagnosed with Fabry disease (1). In our patient, Fabry disease was suggested by the pathologist evaluating kidney biopsy. It took several years to establish the diagnosis at the Department of Rare Diseases in Cracow. In some patients with Fabry disease, a pacemaker is needed due to symptomatic bradycardia. Recent retrospective studies have shown that 1–8 % of Fabry patients require pacemakers implantation [5]. Unfortunately, the use of cardiovascular implantable electronic devices is associated with a risk of infection even in the general population. The reported incidence of infection of cardiac devices ranges from 0.5 % to 5.1 % and is associated with substantial morbidity and mortality. Furthermore, the prognosis is even worse when CDRE occurs. One of the major risk factors of CDRE is kidney disease. Moderate to severe chronic kidney disease is identified as the most potent risk factor for device-related infections. Patients with Fabry disease, as most of the develop end-stage renal failure, are at increased risk of cardiac device-related endocarditis. In our case, CDRE and subsequent treatment with nephrotoxic drugs, including vancomycin, was the reason for significant worsening of renal function requiring the start of renal replacement therapy.
